# Bilateral Patellar Avascular Necrosis Following Total Knee Arthroplasties

**DOI:** 10.7759/cureus.76798

**Published:** 2025-01-02

**Authors:** Glynnis K Page, Jorge Marina, Amy Hoang, Julia Thompson, Lynn Voss

**Affiliations:** 1 Orthopedics, Rocky Vista University College of Osteopathic Medicine, Parker, USA; 2 Orthopedic Surgery, BoulderCentre for Orthopedics and Spine, Boulder, USA

**Keywords:** avascular necrosis (avn), hardware removal, knee total arthroplasty, lateral retinaculum, patella

## Abstract

A 79-year-old female with a history of bilateral knee osteoarthritis presented with severe knee pain, eight years post-bilateral total knee arthroplasties (TKAs). Notably, a lateral retinacular release had been performed alongside each arthroplasty. On examination, she displayed new painful prominences along the lateral aspects of both knees. Radiographs and CT scans revealed near-complete resorption of the bilateral patellae, indicating advanced avascular necrosis (AVN). After discussing treatment options, the patient opted for revision surgery to remove the patellar hardware, leading to significant improvements in pain, function, and mobility. This case underscores the need for close, long-term follow-up in patients who undergo TKA with concurrent lateral retinacular release, to monitor for potential complications like AVN.

## Introduction

Avascular necrosis (AVN), also known as osteonecrosis or ischemic necrosis, is marked by hypoxic injury and subsequent necrosis of osteocytes and bone marrow [1]. While trauma is a common trigger for AVN, several other risk factors, including corticosteroid use, gout, alcoholism, scleroderma, systemic lupus erythematosus, sickle cell disease, and certain genetic predispositions, have been associated with the condition [1,2]. Each year in the United States, approximately 15,000 new AVN cases are diagnosed, contributing to up to 10% of all total joint replacements [2,3].

AVN can affect multiple bony areas, with the greatest risk in regions that depend on a single blood supply, making them more susceptible to compromise [1]. Early-stage AVN often presents with subtle symptoms, primarily from bone marrow edema causing pain [1]. As the condition progresses, subchondral collapse leads to degenerative arthritis and chronic pain associated with critical ischemia [1].

The patella is supplied by an extensive vascular network, including an extraosseous anastomotic ring from the anterior tibial recurrent arteries (supreme, medial, superior, and inferior) and geniculate arteries (lateral, superior, and inferior) [4]. Intraosseous circulation is maintained by mid-patellar and polar vessels [4]. During a total knee arthroplasty (TKA), the medial parapatellar approach is commonly used, which disrupts key arteries such as the descending geniculate and various geniculate branches, leaving only the superolateral geniculate artery intact [5]. If a lateral retinacular release is performed, this last remaining artery is also compromised, eliminating the extraosseous anastomotic ring [5].

Thus, patellar AVN is a significant complication of TKA, particularly when a lateral retinacular release is included, and it warrants vigilant postoperative assessment to ensure early detection and intervention [5,6].

## Case presentation

A 79-year-old female with a history of bilateral TKAs with lateral retinacular releases, performed in 2014 to address osteoarthritis, presented with chronic anterior knee pain, swelling, and new lateral prominences over both knees. She reported that the pain and swelling began a few months post-surgery, making it challenging for her to ambulate without a walker. She also described a grinding sensation within the knee and a decreased range of motion, particularly with knee extension. Despite multiple knee aspirations, she experienced minimal symptomatic relief.

Her past medical history includes GERD and hypothyroidism, with no other chronic conditions, corticosteroid use, or history of alcohol, tobacco, or recreational drug use. On physical examination, both knees exhibited large effusions and limited range of motion. Palpation revealed a noticeable discontinuity across the patellae, with a sharp prominence along the lateral trochlea. Varus and valgus stress testing showed stable collateral ligaments, and the distal neurovascular exam was normal in both lower extremities.

**Figure 1:**
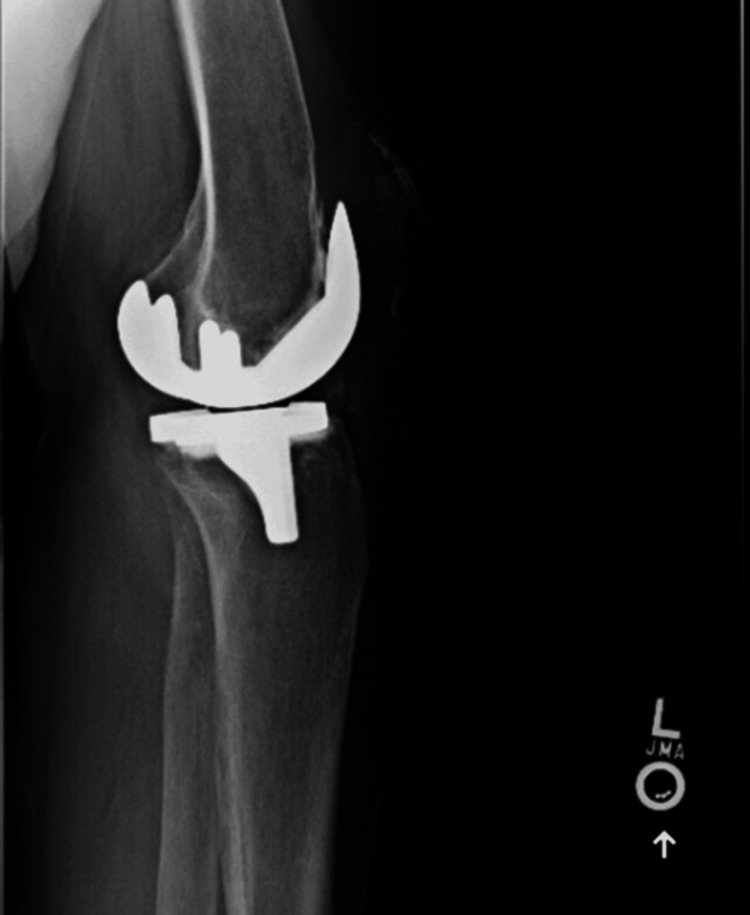
Preoperative lateral radiograph of the left knee demonstrating resorption of the left patella

**Figure 2:**
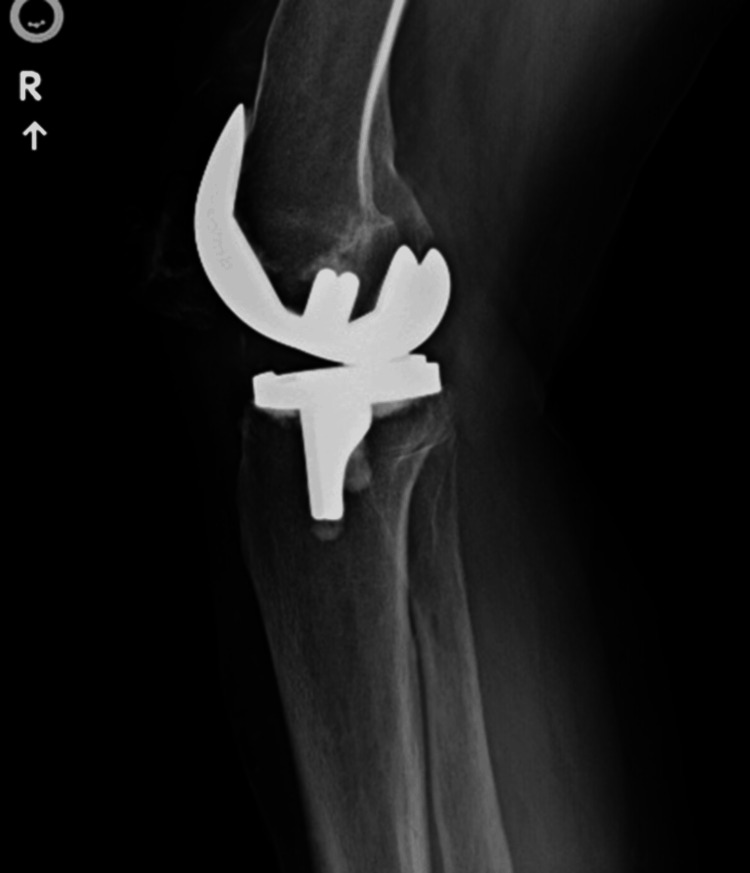
Preoperative lateral radiograph of the right knee demonstrating resorption of the right patella

**Figure 3:**
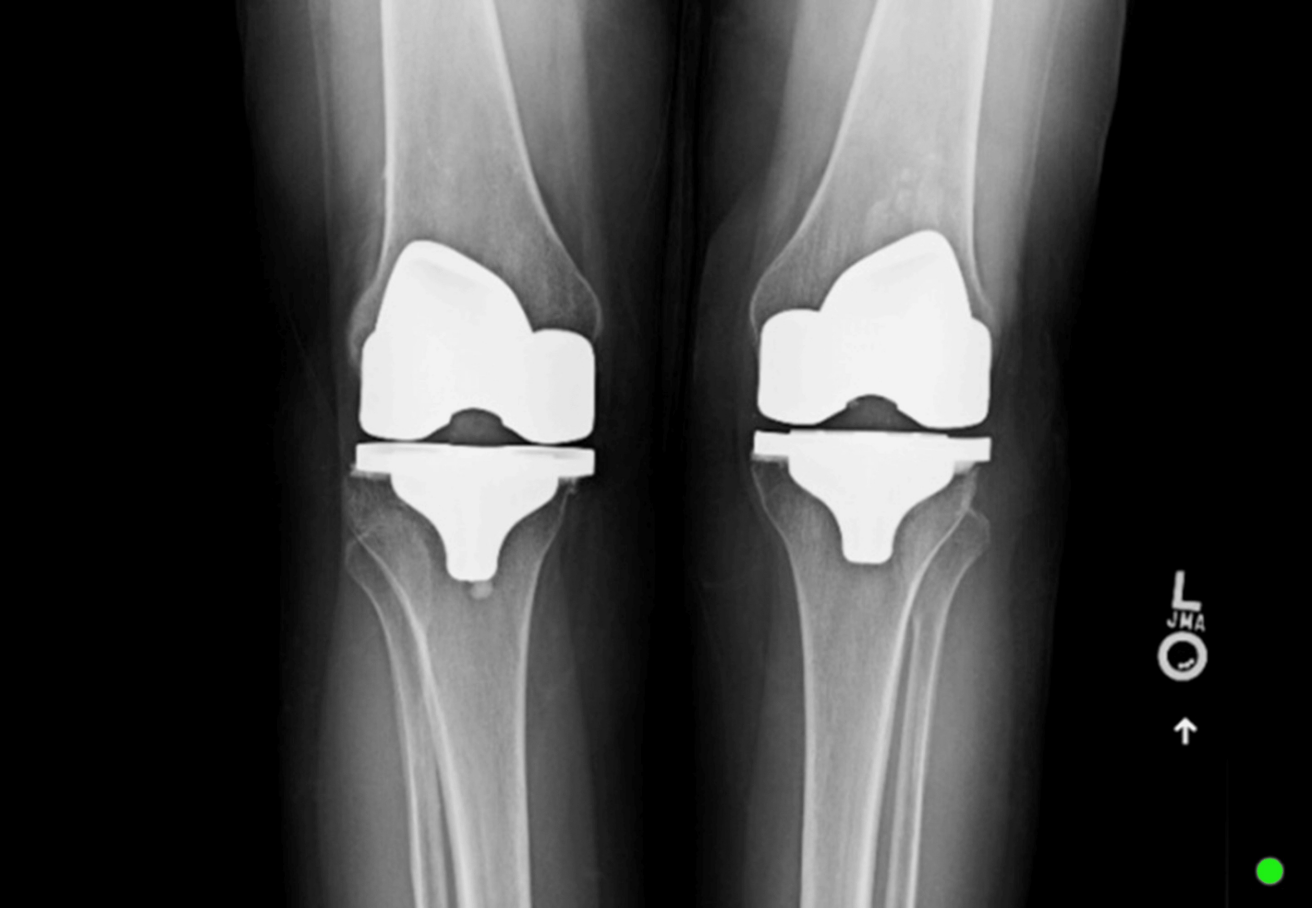
Preoperative anteroposterior radiograph of the bilateral knees

**Figure 4:**
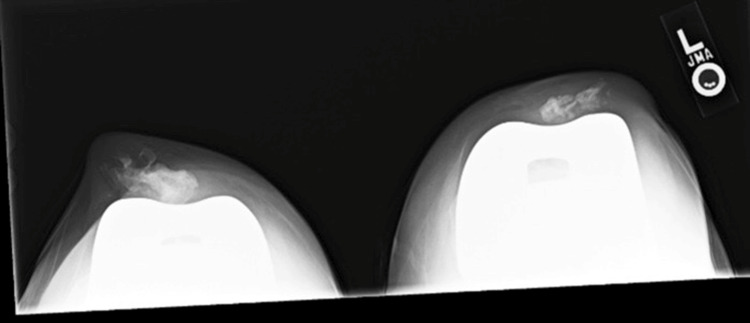
Preoperative sunrise radiograph of bilateral knees demonstrating resorption of both patellae and subluxation of the patellar implant components

Initial X-rays of the bilateral knees (Figure 1, Figure 2, Figure 3, Figure 4) demonstrated near-complete osteolysis of the bilateral patellae, with scattered bony fragments visible in the trochlear grooves. The patellar components appeared partially visible, with lateral subluxation and rotation on the sunrise view.

A subsequent CT scan revealed near-complete resorption of the left patella, leaving only a few small bone fragments, while the right patella retained slightly more bone. Both patellar buttons had migrated anteriorly and laterally with rotational deformities. Due to prosthetic artifacts, X-rays provided a clearer view of these findings.

After reviewing both surgical and nonsurgical treatment options, along with their associated risks and benefits, the patient elected to proceed with patellar component excision and lateral retinacular repair.

**Figure 5:**
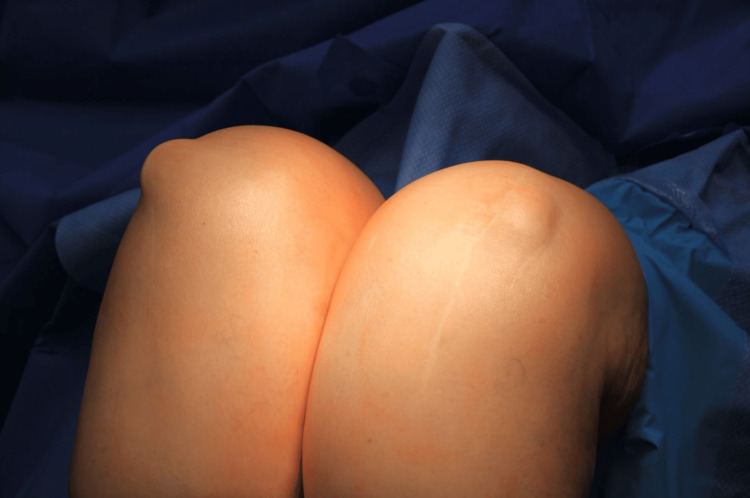
Bilateral knee flexion prior to excision of the patellar component demonstrating subluxation of patellar implants

**Figure 6:**
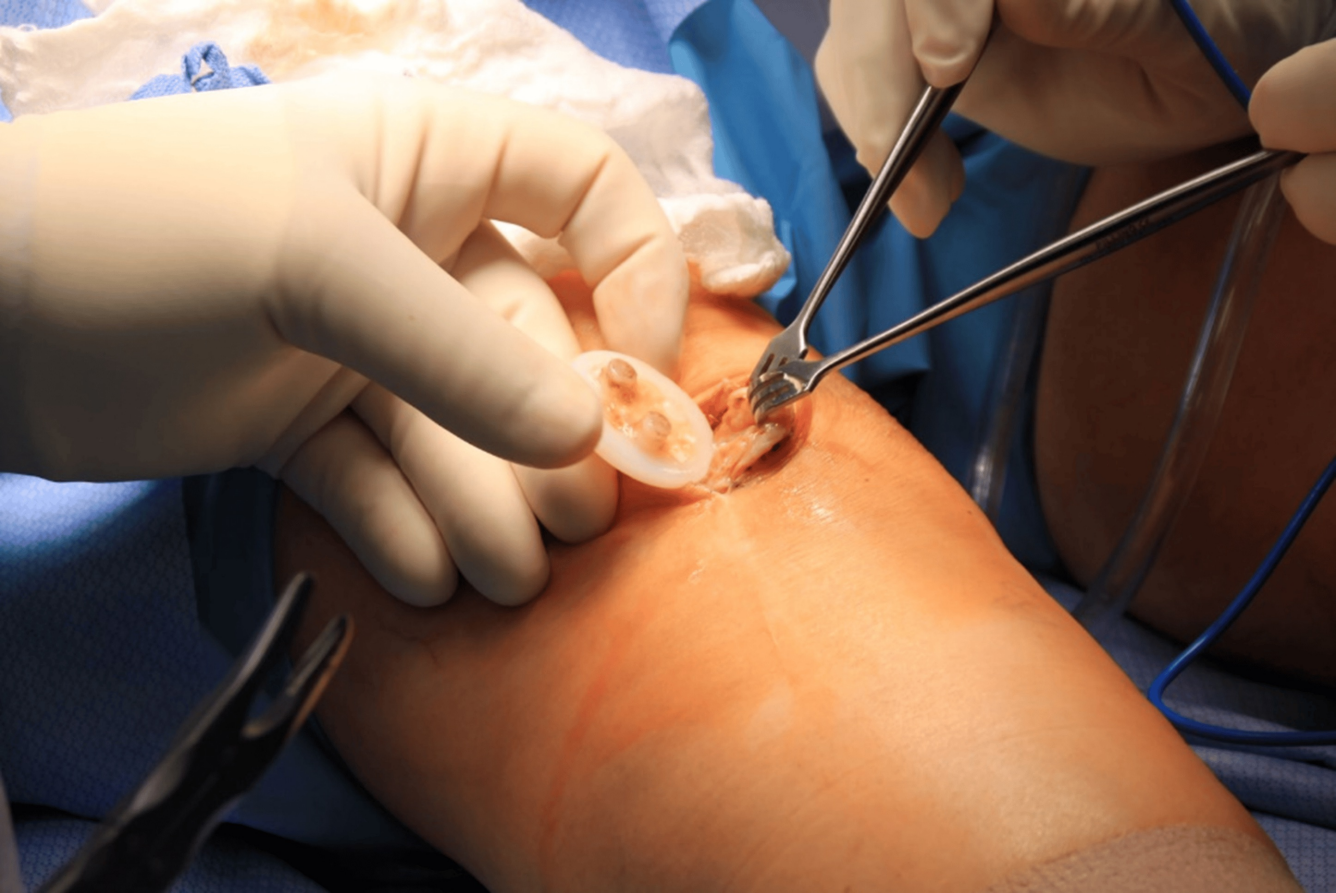
Excision of the patellar component demonstrating lack of native bone, confirming patella resorption seen on radiographs

**Figure 7:**
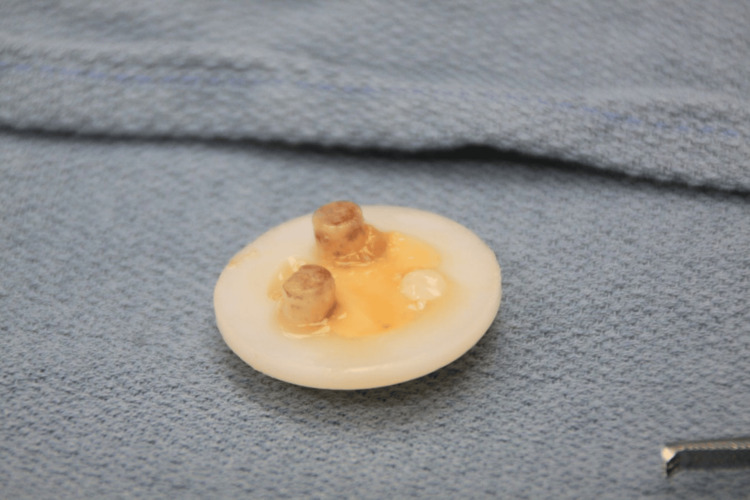
Excised patella component

**Figure 8:**
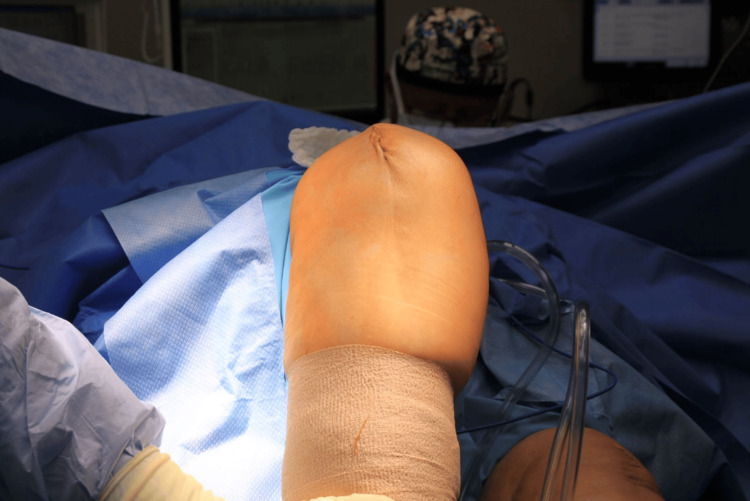
Postoperative knee flexion demonstrating resolution of rotational deformities

In the operating room, bilateral knees were marked as the correct surgical sites. The patient received epidural anesthesia and IV sedation, with tourniquets applied to both thighs, and each leg was prepared with full aseptic technique. Once prepped and draped, the right knee was exsanguinated, and an incision was made along the prior scar over the palpable patella. Sharp dissection through the skin and subcutaneous tissue led to blunt dissection down to the patellar component, noting a rent in the lateral capsule. The patellar component, unattached to soft or bony structures, was removed entirely, and synovitis within the knee was debrided. The wound was irrigated thoroughly with an antibiotic solution and closed in layers. The same procedure was then performed on the left knee. Marcaine was infiltrated around each incision, and the sites were dressed with Steri-Strips and Mepilex. Intraoperative images are shown in Figure 5, Figure 6, Figure 7, and Figure 8.

At the first postoperative visit, the patient reported improved functionality and mobility. However, she noted continued pain and swelling on the right side, along with occasional difficulty clearing her right foot during the swing phase of gait.

On examination, her right knee displayed a well-healed surgical incision, mild effusion, 125 degrees of flexion, 5-/5 extensor mechanism strength, and stability on varus and valgus stress testing. A subtle prominence over the anterior knee, at the former patellar hardware site, remained unchanged with movement. Examination of the left knee showed similar findings, without any noticeable prominence. X-rays of both knees confirmed her status post-bilateral patellectomy and patellar hardware removal, with the appropriate alignment of the femoral and tibial components.

At the two-month postoperative check, the patient was progressing well, and a physical therapy regimen was recommended to enhance gait and quadriceps strength.

Four months postoperatively, she reported no further issues with the right knee but continued to experience pain, swelling, and a significant extensor mechanism deficit on the left side. The evaluation indicated failure of the extensor mechanism repair on the left, necessitating a revision procedure, which was successfully completed five months after the initial repair.

At her final follow-up, the patient reported overall improvement, although she faced challenges finding physical therapy due to her rural location. Examination revealed no notable defects in her quadriceps mechanism.

## Discussion

TKA is a relatively common procedure in the United States, with the American Joint Replacement Registry recording 2,244,587 primary and revision hip and knee arthroplasties between 2012 and 2020, with primary TKA accounting for up to 54.5% of all cases [7]. Patellofemoral complications represent approximately 10% of all TKAs performed each year [8]. Both patient-related and surgical factors contribute to the risk of patellofemoral issues post-TKA. Patient-related risk factors include anatomical variations such as a knee valgus angle greater than 10 degrees or a patellar thickness less than 18 mm [8].

Surgical factors, particularly procedures conducted alongside TKA, also play a role. Studies show that performing a lateral retinacular release during TKA increases the risk of patellar complications, including a 3.8-fold increase in patellar component loosening and a 2.7-fold increase in patellar fractures [9]. This heightened risk is linked to the potential development of patellar AVN, emphasizing the need for careful consideration of lateral retinacular release during TKA [5,7].

AVN can present as either a systemic or localized condition. Systemic AVN is often complex, arising from conditions like epiphyseal necrosis or bone infarction, while local AVN, such as patellar AVN, typically results from trauma or repetitive microtrauma to the bone [9,10]. Catching AVN in its early stages remains challenging, as its delayed presentation is often associated with significant bone tissue necrosis by the time symptoms appear [10]. Although the triggers for AVN are not fully understood, several mechanisms have been proposed, including intraluminal vessel obstruction by fat emboli, caisson disease (nitrogen bubbles from decompression sickness), localized clotting due to procoagulant abnormalities, and sickle cell disease [10]. In general, any disruption to the normal blood supply of bone tissue can initiate AVN [6].

In cases of traumatic AVN, the date of injury or trauma typically marks the onset [9,10]. MRI is particularly useful for early detection, often identifying AVN before it becomes visible on radiographs [9,10]. In the early stages, an interface forms between healthy and necrotic bone, producing a signal on MRI that facilitates early diagnosis [10]. A study on patients with femoral neck fractures found MRI evidence of AVN in 39% of patients within one to six months post-injury, while only 25% showed AVN on radiographs in the same time frame [10]. Typically, AVN develops within six months of trauma or exposure to a risk factor, with spontaneous radiographic improvement being very rare [10].

For AVN of the patella, nonoperative management is common if there is no fracture or implant loosening [11]. However, if the implant becomes loose, surgical removal is often necessary [8].

The lateral retinaculum provides some lateral stability to the patella, but excessive stress on this structure can cause abnormal lateral tilt and maltracking of the patella [11]. Lateral retinacular release is a widely used procedure to treat anterior knee pain due to extensor mechanism dysfunction, patellar instability, or patellofemoral osteoarthritis [11]. However, in the context of TKA, this release can increase the risk of AVN of the patella [6,11-13].

In a study of 27 patients who had undergone TKA with or without concurrent lateral retinacular release, 15% of those without the release and 56.3% of those with it showed reduced patellar blood flow on bone scans [8]. One patient in this study, who had a normal patella before revision surgery, demonstrated a “cold” patella on bone scan post-lateral release, indicating diminished blood flow [8]. This suggests that performing a lateral retinacular release during or after TKA increases the risk of patellar blood flow inhibition by over three-fold, raising the likelihood of AVN [8,9,14].

Further supporting this, a 2008 study found that lateral release resulted in patellar fractures due to osteonecrosis in 5.2% of cases [9]. This highlights the potential for lateral retinacular release to compromise blood flow, leading to complications like AVN.

Multiple factors contributed to the severity of this patient’s bilateral AVN, with limited access to medical resources likely playing a significant role. Rural communities often encounter healthcare barriers, which can delay diagnosis and treatment, worsening conditions over time. Ultimately, the chosen surgical interventions provided meaningful improvement in her quality of life, addressing the limitations and pain caused by advanced AVN.

## Conclusions

Most reported cases of AVN in the patella show only partial bone resorption, whereas this case demonstrated near-complete resorption of both patellae. Although the overall prevalence of AVN following TKA is relatively low, the risk increases significantly when a lateral retinacular release is performed concurrently. Current data suggests that lateral retinacular release may be contraindicated in patients undergoing TKA for this reason. Orthopedic surgeons must be vigilant in monitoring patients with persistent knee pain, swelling, or ambulation difficulties after TKA with a lateral retinacular release, and, ideally, should avoid the procedure altogether in such cases. When AVN progression becomes inevitable, it is the surgeon’s responsibility to ensure that patients receive timely treatment to maintain the best possible quality of life.
